# Malnutrition risk and oropharyngeal dysphagia in the chronic post-stroke phase

**DOI:** 10.3389/fneur.2022.939735

**Published:** 2022-09-28

**Authors:** V. A. L. Huppertz, W. Pilz, G. Pilz Da Cunha, L. C. P. G. M. de Groot, A. van Helvoort, J. M. G. A. Schols, L. W. J. Baijens

**Affiliations:** ^1^Department of Respiratory Medicine, Maastricht University, Maastricht, Netherlands; ^2^School for Nutrition and Translational Research in Metabolism (NUTRIM), Maastricht University, Maastricht, Netherlands; ^3^Department of Otorhinolaryngology, Head and Neck Surgery, Maastricht University Medical Center, Maastricht, Netherlands; ^4^School for Oncology and Reproduction (GROW), Maastricht University, Maastricht, Netherlands; ^5^School for Mental Health and Neuroscience (MHeNs), Maastricht University, Maastricht, Netherlands; ^6^Division of Human Nutrition and Health, Wageningen University and Research Centre, Wageningen, Netherlands; ^7^Danone Nutricia Research, Utrecht, Netherlands; ^8^Department of Health Services Research, Maastricht University, Maastricht, Netherlands; ^9^Care and Public Health Research Institute (CAPHRI), Maastricht University, Maastricht, Netherlands; ^10^Department of Family Medicine, Maastricht University, Maastricht, Netherlands

**Keywords:** malnutrition, dysphagia, chronic post-stroke, malnutrition screening, dysphagia severity, nutritional risk

## Abstract

**Background:**

Oropharyngeal dysphagia (OD) and malnutrition are associated with poor clinical outcomes after stroke. The present study evaluated (1) malnutrition risk and OD-related characteristics in patients with chronic post-stroke OD, and (2) the relationship between on the one hand OD severity and on the other hand functional oral intake and dysphagia-specific quality of life.

**Methods:**

A cross-sectional study was conducted in a Dutch interdisciplinary outpatient clinic for OD. The standardized examination protocol comprised: clinical ear, nose, and throat examination, body mass index, the short nutritional assessment questionnaire (SNAQ), a standardized fiberoptic endoscopic evaluation of swallowing (FEES), the functional oral intake scale (FOIS), and the MD Anderson dysphagia inventory (MDADI).

**Results:**

Forty-two consecutive patients with chronic post-stroke OD were included. Mean (±SD) age and BMI of the population were 69.1 (±8.7) years and 26.8 (±4.1) kg/m^2^ respectively. Seventeen (40.4%) patients presented a moderate to high risk of malnutrition (SNAQ score≥2). The FEES examination showed moderate to severe OD in 28 (66.7%) patients. The severity of OD was significantly related to the FOIS score but not to the MDADI scores.

**Conclusion:**

In this specific sample of referred stroke patients, moderate to severe OD and moderate to high risk of malnutrition were common. Despite the use of clinical practice guidelines on stroke and a normal nutritional status at first sight, repeated screening for malnutrition and monitoring the severity and management of OD remain important elements in the care of patients with chronic post-stroke OD.

## Introduction

Stroke is ranked within the top ten of diseases that increase the global burden of disease in adults (≥25 years) (1). Current stroke treatments including reperfusion strategies are effective (2, 3), increase the chance of survival, and reduce the rate of disability after stroke (4–6). Yet, these treatments do not guarantee full recovery after stroke and so the demand for rehabilitation and long-term care rises given the increasing number of stroke survivors. Common detrimental clinical outcomes after stroke are functional and cognitive impairment (7–9), disability (10), malnutrition (11–13), skeletal muscle mass loss (14), oropharyngeal dysphagia (OD), altered systemic immunity, and systemic illnesses such as aspiration pneumonia (15–18).

The extent and pace at which stroke patients recover depend on multiple factors, including nutritional status. Nutritional deficiency is a significant contributing factor to impaired functional outcome (19, 20), post-stroke complications, cognitive impairment, and mortality (21–23). Malnutrition is a multidimensional concept in stroke care as it can be both, a cause and a consequence of cognitive- and functional problems after stroke. Cognition and functionality of the swallowing mechanism are conjointly important for the intake of nutrition. Swallowing involves multiple muscles and nerves that rely on the central nervous system for sensory feedback, motor programming and execution, and cognitive cortical processing (24–27). Damaged brain tissue after stroke may result in impaired sensory and motor mechanisms that are essential for swallowing. It is also known that stroke patients often already have a pre-stroke sedentary lifestyle (28), resulting in a higher risk of sarcopenia (29). Moreover, catabolic pathways of muscle tissue are activated after stroke causing loss of skeletal muscle mass which may affect not only peripheral skeletal muscles, but hypothetically also the muscles involved in swallowing (30–34). Severe loss of skeletal muscle mass can cause or enhance swallowing difficulties. OD is one of the factors leading to reduced oral food intake in stroke patients (35). This reduced oral food intake in turn contributes to malnutrition and loss of skeletal muscle mass, thus completing the vicious circle. Swallowing impairment or OD is the difficulty in bolus preparation, airway protection, and/or bolus transport from the mouth to the esophagus. OD may increase the risk of aspiration pneumonia and mortality in stroke patients (36–39). Adequate intake and absorption of nutrients is important, especially in stroke patients, as these may enhance functional recovery (40, 41), brain tissue repair, prevent cognitive decline, and strengthen the immune system (42, 43). Stroke patients however are known to have a diminished energy and protein intake (40). The risk of malnutrition was shown to increase 2.6-fold in stroke patients with OD (44).

Furthermore, the literature showed that OD may affect patients' health-related quality of life (QoL) and wellbeing due to dietary modifications, anxiety, tube feeding dependency, fear of choking, and embarrassment to eat in public, etc. (45–49). The prevalence of OD varied between healthcare settings and was reported in up to 80% of stroke patients across healthcare settings (50). A few stroke patients developed OD within 6 months after stroke or suffered from persistent OD at 6 months or longer after stroke (50, 51). A recent cost-of-illness study found that the healthcare costs during hospitalization of dysphagic stroke patients significantly increased as compared to stroke patients without OD. The same study showed that the OD-related complications malnutrition and respiratory tract infections were associated with an exponential increase in healthcare costs within 1 year after stroke onset (52).

Stroke care in patients with risk of malnutrition and OD is complicated due to the multidimensional causes and poor clinical outcomes of both conditions. An interdisciplinary clinical approach targeted at the identification, diagnosis, and treatment of malnutrition and OD may improve stroke care and clinical outcomes after stroke.

Research on nutritional and OD-related characteristics of patients with chronic post-stroke OD is needed to increase the body of evidence for best-clinical-practice stroke care and clinical guidelines. Studies showed that there are many unmet needs at various domains of health and healthcare for long-term stroke survivors (53) and that life after stroke is an understudied area in stroke research that requires attention also in national stroke plans in the future (54). Literature reviews on nutritional status in stroke patients across the continuum of care showed a lack of studies and also a limited number of studies aiming at both, nutritional status and OD in the late subacute and chronic phase after stroke (55, 56). In the first place, the present study determined the prevalence and severity of nutritional risk and OD-related characteristics in patients with chronic post-stroke OD using the short nutritional assessment questionnaire (SNAQ) and a fiberoptic endoscopic evaluation of swallowing (FEES). Secondly, the relationship between on the one hand OD severity and on the other hand functional oral intake and dysphagia-specific QoL was explored.

## Methods

### Study design and study population

This exploratory cross-sectional study enrolled consecutive patients with chronic post-stroke (≥6 months) OD who visited the interdisciplinary outpatient clinic for OD in a tertiary university referral hospital in the Netherlands between 2013 and 2020. The population of patients with OD in the late subacute and chronic phase after stroke is not easily approachable in the Netherlands. Patients stay in various settings: at home, in a nursing home, in a residential care center, etc. It is likely that this diversity of care settings also leads to heterogeneous care and few patients with chronic OD find their way to specialized care resulting in this 7-year period of recruitment.

Patients with clinically relevant dysphagic complaints, as indicated by their referring speech-and-language therapist from the primary healthcare network were included in the present study. Inclusion criteria were based on a wide spectrum of swallowing complaints such as among others, difficulties in oropharyngeal bolus formation and transit, slow eating, coughing while drinking or eating, history of aspiration pneumonia, abnormal amounts of residue in the oral cavity, choking, abnormal gaging during intake, weight loss etc.

Exclusion criteria were a diagnosis other than stroke that could cause OD, a score below 23 on a Mini Mental State Examination (57), and illiteracy or blindness. In the present study, no patients were excluded based on these criteria.

A standardized examination protocol, used in daily clinical practice at the outpatient clinic, was carried out. This prospectively standardized protocol comprised a clinical ear, nose, and throat examination (including cranial nerve function) performed by a laryngologist, determination of body mass index (BMI), the Functional Oral Intake Scale (FOIS) (58), the MD Anderson Dysphagia Inventory (MDADI) (59–61), the SNAQ (62, 63), and a standardized FEES (64–66).

### Ethical considerations

The current study was approved by the ethical committee according to the Medical Research Involving Human Subjects Act (Wet Medisch Wetenschappelijk Onderzoek [WMO]) as non-WMO research (METC 2021-2519O) (67). The no-objection system for the use of patient data for scientific research was applied and data were completely pseudonymized.

### Data collection

Data were collected in a standardized way by a laryngologist and a speech-and-language therapist as part of regular care. This concerned data on patient demographics (gender, age, BMI), medical history (recurrent stroke, date of last stroke event, speech-and-language therapy), primary objectives (nutritional risk and OD severity), and exploratory objectives (functional oral intake, dysphagia-specific QoL).

### Primary objectives

Nutritional risk was examined using the original SNAQ malnutrition questionnaire, a validated screening tool to identify patients at risk of malnutrition in the hospital outpatient setting (62, 63). The SNAQ is a self-reported questionnaire consisting of three questions on unintended weight loss, decreased appetite, and the use of oral nutritional supplementation or tube feeding. The answers were dichotomous (yes/no) and resulted in a total score indicating the risk for malnutrition. A score below two points indicated a low risk for malnutrition, a score of two points indicated a moderate risk for malnutrition, and a score of three points or more indicated a severe risk for malnutrition. Patients with a SNAQ score of three points or more were referred to a dietitian for a detailed nutritional assessment.

To identify the characteristics and severity of OD, each patient underwent a standardized FEES. The FEES protocol consisted of three trials of thin liquid (3 x 10 ml water), three trials of thick liquid (3 x 10 ml applesauce; One 2 fruit), and one trial of a bite-sized cracker (Delhaize mini toast 80 gr). To enhance endoscopic visualization of the bolus, water and applesauce were dyed with 5% methylene blue 10 mg/ml (65, 66). The viscosities of thin and thick liquid boluses were respectively 1 mPa and 1,200 mPa per second measured at 25 degrees Celsius 50 s−1 of shear rate as recommended by the National Dysphagia Diet (68). According to the International Dysphagia Diet Standardization Initiative (IDDSI), thin liquid met the criteria for IDDSI level zero (thin) and thick liquid for level three (moderately thick)(69). The position of the tip of the flexible endoscope (Pentax FNL-10RP3, Pentax Canada Inc., Mississauga, Ontario, Canada) allowed observation of the pharyngeal and laryngeal anatomy and physiology during the pharyngeal phase of swallowing. Topical anesthetics were not used as they may affect pharyngolaryngeal sensory function. FEES videos were recorded at 25 frames per second using a Xion SD camera, XionEndoSTROB E camera control unit and Matrix DS data station with DIVAS software (Xion Medical, Berlin, Germany) and the data were stored in a secured drive on the hospital network.

For each bolus swallow, the visuoperceptual variables, penetration-aspiration, pharyngeal residue, and ‘other signs' of OD (pre-swallow posterior spill, delayed initiation of the pharyngeal reflex, piecemeal deglutition) were assessed di- or trichotomously by two observers (laryngologist and a speech-and-language therapist) using consensus agreement (Supplementary Table S1) (66, 70, 71). For the present study, consensus agreement by two experienced observers was chosen, as this method showed a better reproducibility of measurements in terms of observer agreement compared to the independent rating method, as discussion on measurements in a panel improved concordance in previous studies (65, 66, 72).

The severity of OD was evaluated using the Dysphagia Severity Scale (DSS) by Dziewas et al. (73–75). A DSS score of zero points was defined as the absence of clinically relevant OD. A score of one point indicated mild OD defined by the presence of premature spillage and/or pharyngeal residue, but without penetration-aspiration events. A DSS score of two points indicated moderate OD defined by the presence of penetration-aspiration events with one bolus consistency. A DSS score of three points indicated severe OD defined by the occurrence of penetration-aspiration with two or more bolus consistencies.

### Exploratory objectives

To explore the relationship between OD severity and respectively functional oral intake (the level of oral or non-oral intake) and dysphagia-specific QoL, the FOIS (58) and the Dutch version of the MDADI for neurogenic OD were used (59). The FOIS was completed by the clinician during a structured interview. Patients with a FOIS score of three or lower were completely or partially tube dependent. Patients were completely tube dependent in case oral food intake was not recommended (FOIS 1) or if minimal or inconsistent oral intake was possible (FOIS 2). Patients were partially tube dependent if additional tube supplements were required next to a consistent oral intake of food or liquid (FOIS 3). Patients with a FOIS score above three did not require tube feeding and total oral intake was possible with or without texture modifications and/or thickening of liquids. A patients' diet may have been restricted to a single consistency (FOIS 4) or to multiple consistencies with special preparation (FOIS 5). Diet restrictions may also have been limited to the elimination of specific foods or liquid items only (FOIS 6) or to no restrictions at all (FOIS 7).

The MDADI is a self-report questionnaire to measure the impact of OD on health-related QoL and the Dutch version has also been validated for patients with neurogenic OD (61). The MDADI consists of different domains: one global assessment question (MDADI-G) for the effect of OD on overall health-related QoL; the functional scale (MDADI-F) for the impact of OD on daily activities (five questions); the physical scale (MDADI-P) for the physical impact of OD as perceived by the patient (eight questions); and the emotional scale (MDADI-E) for the patients' perceptual response on OD, e.g. self-consciousness, embarrassment, etc. (six questions). The questions were scored on a five-point Likert scale (1=strongly agree/2=agree/3=no opinion/4=disagree/5=strongly disagree) and the MDADI total score (MDADI-T) was based on the sum of all domains (20 questions). The minimum score was 20 representing a poor dysphagia-specific QoL and the maximum possible score is 100 (high dysphagia-specific QoL).

### Statistical analyses

Statistical analyses were performed in IBM SPSS statistics 25 (IBM SPSS Statistics, IBM Corporation, Chicago, IL). Normality of continuous variables was determined with QQ-plots and the Shapiro Wilk test. It was checked whether missing data were missing at random. Thereafter, pairwise deletion could be used for the analyses to minimize data loss. Fisher's exact test was conducted to check for differences in categorical variables between groups. For statistical purposes, trichotomous data on penetration-aspiration were dichotomized into zero for the absence of penetration and aspiration and one for penetration or aspiration. Differences in mean for continuous variables between groups were analyzed using the independent sample *t*-test. The non-parametric independent Mann-Whitney U test was used to compare median values and non-normally distributed continuous variables. Univariable and multivariable binary logistic regression analyses were performed to assess the relationship between OD severity (DSS) and respectively functional oral intake (FOIS) and patients' dysphagia-specific QoL (MDADI). Independent variables in multivariable regression analyses were limited to two variables per model due to the low number of events per variable (76). To control for confounding, corrections were applied in multivariable regression analyses.

## Results

The study sample consisted of 42 patients with chronic post-stroke (≥6 months) OD. The sample consisted of 32 (76.2%) male patients. Mean (±SD) age and BMI of the total population were 69.1 (±8.7) years and 26.8 (±4.1) kg/m^2^ respectively. Eleven (26.2%) patients suffered from recurrent stroke and the mean (±SD) time since the last stroke event in the total sample was 39.3 (±50.8) months. Twenty-seven (64.3%) patients received speech-and-language therapy after the stroke event. Details on patient demographics and medical history can be found in Table 1.

**Table 1:** Patient characteristics of the total population (*n* = 42) and in patients with low risk of malnutrition vs. moderate or high risk of malnutrition according to the SNAQ (*n* = 41).

	**Total population (*n =* 42)**	**Low malnutrition risk (*n =* 24)**	**Moderate/high malnutrition risk (*n =* 17)**	
***Patient demographics***	***n (%)***	***n (%)***	***n (%)***	***p-value***
Gender				
• *Female*	10 (23.8)	5 (20.8)	5 (29.4)	0.714
• *Male*	32 (76.2)	19 (79.2)	12 (70.6)	
Age (years) [*mean* (±*SD*)]	69.1 (±8.7)	70.6 (±8.3)	66.5 (±9.1)	0.047^a*^
Body Mass Index (kg/m^2^) [*mean* (±*SD*)]	26.8 (±4.1)	27.0 (±3.7)	26.6 (±4.8)	0.757
• *Missing value*	2	1	1	
Body Mass Index categories				
• * < 18.5 kg/m^2^*	0 (0.0)	0 (0.0)	0 (0.0)	1.000
• *18.5-24.9 kg/m^2^*	16 (38.1)	9 (39.1)	6 (37.5)	
• *25.0-29.9 kg/m^2^*	13 (31.0)	8 (34.8)	5 (31.3)	
• *≥30 kg/m^2^*	11 (26.2)	6 (26.1)	5 (31.3)	
• *Missing value*	2	1	1	
***Medical history***	***n (%)***	***n (%)***	***n (%)***	***p-value***
Recurrent stroke				
• *None*	31 (73.8)	20 (83.3)	10 (58.8)	0.241
• *One*	8 (19.0)	3 (12.5)	5 (29.4)	
• *Two*	2 (4.8)	1 (4.2)	1 (5.9)	
• *Three*	0 (0.0)	0 (0.0)	0 (0.0)	
• *Four*	1 (2.4)	0 (0.0)	1 (5.9)	
Time (months) since last stroke event [*mean* (±*SD*)]	39.3 (±50.8)	28.7 (±43.0)	52.0 (±60.0)	0.165
• *Missing value*	2	1	1	
Time since last stroke event				
• * ≤ 24 months*	22 (52.4)	14 (60.9)	8 (50.0)	0.531
• *>24 months*	18 (42.9)	9 (39.1)	8 (50.0)	
• *Missing value*	2	1	1	
Speech-and-language therapy				
• *No*	15 (35.7)	9 (37.5)	5 (29.4)	0.742
• *Yes*	27 (64.3)	15 (62.5)	12 (70.6)	
***Primary objectives***	***n (%)***	***n (%)***	***n (%)***	***p-value***
Unintended weight loss >6 kg				
• *No*	28 (66.7)	24 (100.0)	4 (26.7)	< 0.001**
• *Yes*	11 (26.2)	0 (0.0)	11 (73.3)	
• *Missing value*	3	0	2	
Unintended weight loss >3 kg				
• *No*	32 (76.2)	24 (100.0)	8 (53.3)	< 0.001**
• *Yes*	7 (16.7)	0 (0.0)	7 (46.7)	
• *Missing value*	3	0	2	
Decreased appetite				
• *No*	28 (66.7)	18 (75.0)	10 (66.7)	0.718
• *Yes*	11 (26.2)	6 (25.0)	5 (33.3)	
• *Missing value*	3	0	2	
Oral nutritional supplement or tube feeding				
• *No*	25 (59.5)	17 (70.8)	8 (53.3)	0.318
• *Yes*	14 (33.3)	7 (29.2)	7 (46.7)	
• *Missing value*	3	0	2	
Short Nutritional Assessment Questionnaire (SNAQ)				
• *Low*	24 (57.1)	-	-	
• *Moderate*	3 (7.1)	-	-	
• *High*	14 (33.3)	-	-	
• *Missing value*	1			
Penetration				
• *No*	12 (28.6)	6 (26.1)	5 (29.4)	0.141
• *Thin*	15 (35.7)	11 (47.8)	4 (23.5)	
• *Thick*	1 (2.4)	1 (4.3)	0 (0.0)	
• *Solid*	0 (0.0)	0 (0.0)	0 (0.0)	
• *Thin + thick*	9 (21.4)	5 (21.7)	4 (23.5)	
• *Thin + solid*	1 (2.4)	0 (0.0)	1 (5.9)	
• *Thick + solid*	0 (0.0)	0 (0.0)	0 (0.0)	
• *Thin + thick + solid*	3 (7.1)	0 (0.0)	3 (17.6)	
• *Missing value*	1	1	0	
Aspiration				
• *No*	24 (57.1)	16 (66.7)	7 (41.2)	0.105
• *Thin*	14 (33.3)	7 (29.2)	7 (41.2)	
• *Thick*	1 (2.4)	1 (4.2)	0 (0.0)	
• *Solid*	0 (0.0)	0 (0.0)	0 (0.0)	
• *Thin + thick*	2 (4.8)	0 (0.0)	2 (11.8)	
• *Thin + solid*	1 (2.4)	0 (0.0)	1 (5.9)	
• *Thick + solid*	0 (0.0)	0 (0.0)	0 (0.0)	
• *Thin + thick + solid*	0 (0.0)	0 (0.0)	0 (0.0)	
Pharyngeal residue				
• *No*	21 (50.0)	12 (50.0)	8 (47.1)	1.000
• *Yes*	21 (50.0)	12 (50.0)	9 (52.9)	
‘Other signs' of OD				
• *No*	8 (19.0)	6 (25.0)	1 (5.9)	0.207
• s*Yes*	34 (81.0)	18 (75.0)	16 (94.1)	
Dysphagia Severity Scale (DSS)				
• *No relevant OD*	9 (21.4)	4 (18.2)	4 (23.5)	0.325
• *Mild OD*	3 (7.1)	2 (9.1)	1 (5.9)	
• *Moderate OD*	15 (35.7)	11 (50.0)	4 (23.5)	
• *Severe OD*	13 (31.0)	5 (22.7)	8 (47.1)	
• *Missing value*	2	2	0	
***Exploratory objectives***	***n (%)***	***n (%)***	***n (%)***	***p-value***
Functional Oral Intake Scale (FOIS)				
• *FOIS 1*	4 (9.5)	3 (13.0)	1 (6.3)	0.689
• *FOIS 2*	1 (2.4)	0 (0.0)	1 (6.3)	
• *FOIS 3*	0 (0.0)	0 (0.0)	0 (0.0)	
• *FOIS 4*	2 (4.8)	1 (4.3)	1 (6.3)	
• *FOIS 5*	13 (31.0)	6 (26.1)	7 (43.8)	
• *FOIS 6*	7 (16.7)	4 (17.4)	2 (12.5)	
• *FOIS 7*	13 (31.0)	9 (39.1)	4 (25.0)	
• *Missing value*	2	1	1	
	***Median (25th−75 th percentile)***	***Median (25th−75 th percentile)***	***Median (25th−75 th percentile)***	***p-value***
MD Anderson Dysphagia Inventory (MDADI)				
• *Total*	60.0 (53.0-77.0)	61.0 (55.5-78.5)	58.0 (45.0-71.0)	0.499
• *Missing value*	5	3	2	
• *Global*	2.0 (1.0-4.0)	2.5 (1.8-4.0)	2.0 (1.0-4.0)	0.453^a^
• *Missing value*	3	2	1	
• *Functional*	18.0 (14.0-22.0)	18.5 (15.5-22.3)	16.5 (13.3-19.75)	0.664
• *Missing value*	3	1	1	
• *Physical*	23.0 (18.5-28.0)	24.0 (17.0-33.0)	23.0 (19.5-27.0)	0.713
• *Missing value*	1	0	0	
• *Emotional*	19.0 (15.3-22.0)	19.0 (16.0-22.0)	19.0 (13.5-21.8)	0.940
• *Missing value*	2	0	1	

^*a*^ Whitney U-test.

*p < 0.05; **p < 0.001.

### Prevalence of nutritional risk

The SNAQ was completed by 41 patients. The SNAQ score of one patient was missing, as this patient was not willing or able to complete the questionnaire. Results from the SNAQ revealed unintentional weight loss >6 kg in the past 6 months in 11 (26.2%) patients. Unintentional weight loss of >3 kg in the past month was present in seven (16.7%) patients. During the month prior to the visit to the outpatient clinic, eleven (26.2%) patients experienced decreased appetite and 14 (33.3%) patients used oral nutritional supplements (ONS) or tube feeding. Twenty-four (57.1%) patients presented a low risk of malnutrition, three (7.1%) a moderate risk, and 14 (33.3%) a high risk. Details on demographics, medical history, primary and exploratory objectives in patients with low vs. moderate or high risk of malnutrition can be found in Table 1.

Medical history and patient demographics, except for age (*p* = 0.047), did not significantly differ between the two risk-groups, patients with a low risk vs. patients with a moderate or high risk of malnutrition. The prevalence of patients with unintended weight loss of >6 kg in the last 6 months or >3 kg in the last month was significantly different between the nutritional risk groups (*p* < 0.001). The prevalence of patients who experienced a decreased appetite or used ONS or tube feeding did not significantly differ between the nutritional risk groups.

None of the 24 patients with low risk of malnutrition had unintentional weight loss of >6 kg in 6 months or >3 kg in 1 month. Respectively, eleven (73.3%) and seven (46.7%) patients in the high-risk group had unintentional weight loss of >6 kg in 6 months or >3 kg in 1 month.

No significant difference in mean BMI was found between patients with vs. without decreased appetite (*p* = 0.154), or between patients who used ONS or tube feeding vs. patients who did not (*p* = 0.129).

There was no significant difference in the use of ONS or tube feeding between patients with vs. without unintentional weight loss (*p* = 0.156 and *p* = 0.686). ONS or tube feeding was used by six (54.5%) out of 11 patients that lost >6 kg in 6 months and three (42.9%) out of seven patients that lost >3 kg in 1 month.

### Characteristics and the prevalence of different OD severity levels

FEES measurements revealed that 29 (69.0%) of the patients presented penetration, 18 (42.9%) aspiration, 21 (50.0%) pharyngeal residue, and 34 (81.0%) ‘other signs' of OD. Penetration and aspiration predominantly occurred during the swallows of thin liquids, in 14 (35.7%) and 14 (33.3%) patients, respectively.

The OD severity was classified in 40 patients based on the FEES results. The OD severity of two patients could not be retrieved from the FEES results. As shown in Table 1, nine (21.4%) patients did not have clinically relevant OD, three (7.1%) patients had mild OD, 15 (35.7%) patients had moderate OD, and 13 (31.0%) patients were severely dysphagic according to the DSS. However, four (44.4%) of the nine patients without clinically relevant OD (DSS score of 1), did show ‘other signs' of OD in the present study.

Significant differences were not found in patient demographics, medical history or indicators of nutritional risk between patients with no/mild OD vs. patients with moderate/severe OD according to the DSS (Table 2).

**Table 2:** Patient characteristics in patients with no/mild OD severity vs. moderate or severe OD according to the DSS (*n* = 40).

	**DSS no/mild (*n =* 12)**	**DSS moderate/severe (*n =* 28)**	
***Patient demographics***	***n (%)***	***n (%)***	***p-value***
Gender			
• *Female*	3 (25.0)	7 (25.0)	1.000
• *Male*	9 (75.0)	21 (75.0)	
Age (years) [*mean* (±*SD*)]	69.5 (±7.7)	68.8 (±9.1)	0.814
Body Mass Index (kg/m^2^) [*mean (*±*SD)]*	27.8 (±5.1)	26.6 (±3.6)	0.398
Body Mass Index Categories			
• * < 18.5 kg/m^2^*	0 (0.0)	0 (0)	0.614
• *18.5*–*24.9 kg/m^2^*	4 (33.3)	11 (42.3)	
• *25.0*–*29.9 kg/m^2^*	3 (25.0)	9 (34.6)	
• *≥30.0 kg/m^2^*	5 (41.7)	6 (23.1)	
• *Missing value*	0	2	
***Medical history***	***n (%)***	***n (%)***	***p-value***
Recurrent stroke			
• *None*	9 (75.0)	20 (71.4)	1.000
• *One*	3 (25.0)	5 (17.9)	
• *Two*	0 (0.0)	2 (7.1)	
• *Three*	0 (0.0)	0 (0.0)	
• *Four*	0 (0.0)	1 (3.6)	
Time (months) since last stroke event [*mean (*±*SD)]*	62.6 (±63.2)	30.6 (±44.7)	0.086
• *Missing value*	1	1	
Time since last stroke event			
• * ≤ 24 months*	4 (36.4)	17 (63.0)	0.167
• *>24 months*	7 (63.6)	10 (37.0)	
• *Missing value*	1	1	
Speech-and-language therapy			
• *No*	7 (58.3)	8 (28.6)	0.091
• *Yes*	5 (41.7)	20 (71.4)	
***Primary objectives***	***n (%)***	***n (%)***	***p-value***
Unintended weight loss >6 kg			
• *No*	6 (60.0)	20 (74.1)	0.442
• *Yes*	4 (40.0)	7 (25.9)	
• *Missing value*	2	1	
Unintended weight loss >3 kg			
• *No*	7 (70.0)	23 (85.2)	0.360
• *Yes*	3 (30.0)	4 (14.8)	
• *Missing value*	2	1	
Decreased appetite			
• *No*	6 (60.0)	20 (74.1)	0.442
• *Yes*	4 (40.0)	7 (25.9)	
• *Missing value*	2	1	
Oral nutritional supplement or tube feeding			
• *No*	7 (70.0)	18 (66.7)	1.000
• *Yes*	3 (30.0)	9 (33.3)	
• *Missing value*	2	1	
Short Nutritional Assessment Questionnaire (SNAQ)			
• *Low*	6 (54.5)	16 (57.1)	1.000
• *Moderate*	1 (9.1)	2 (7.1)	
• *High*	4 (36.4)	10 (35.7)	
• *Missing value*	1	0	
***Exploratory objectives***	***n (%)***	***n (%)***	***p-value***
Functional Oral Intake Scale (FOIS)			
• *FOIS 1*	0 (0.0)	2 (7.4)	0.031*
• *FOIS 2*	0 (0.0)	1 (3.7)	
• *FOIS 3*	0 (0.0)	0 (0.0)	
• *FOIS 4*	0 (0.0)	2 (7.4)	
• *FOIS 5*	1 (9.1)	12 (44.4)	
• *FOIS 6*	5 (45.5)	2 (7.4)	
• *FOIS 7*	5 (45.5)	8 (29.6)	
• *Missing value*	1	1	
	***Median (25th−75 th percentile)***	***Median (25th−75 th percentile)***	***p-value***
MD Anderson Dysphagia Inventory (MDADI)			
• *Total*	78.0 (51.5–87.5)	58.0 (53.0–71.0)	0.441
• *Missing value*	3	1	
• *Global*	4.0 (2.0–5.0)	2.0 (1.0–4.0)	0.182^a^
• *Missing value*	1	1	
• *Functional*	21.0 (17.0–25.0)	16.5 (14.0–19.8)	0.133
• *Missing value*	2	0	
• *Physical*	24.5 (16.5–31.8)	23.5 (19.5–27.8)	0.730
• *Missing value*	0	0	
• *Emotional*	22.0 (15.0–24.0)	19.0 (15.3–20.8)	0.310
• *Missing value*	1	0	

^*a*^ Whitney U–test.

*p < 0.05; ** p < 0.001.

In four (9.5%) patients, the severity of the patients' OD did not allow the testing of all three bolus consistencies during the standardized FEES. In these cases, the swallow of a solid bolus and/or thin liquid was expected to be clinically unsafe with a high risk of very severe aspiration. This expectation was based on the observation of severe aspiration of saliva at the start of the FEES and/or severe aspiration during the thick liquid bolus swallow.

### The relationship between on the one hand OD severity and on the other hand functional oral intake and dysphagia-specific QoL

A significant difference in FOIS scores was found between the DSS groups (*p* = 0.031). None of the patients in the no/mild OD group presented a FOIS ≤ 4, one (9.1%) patient of this group presented FOIS 5, and10 patients (91.0%) presented a FOIS ≥6. Five (18.5%) patients in the moderate/severe OD group presented a FOIS ≤ 4, twelve (44.4%) patients presented FOIS 5, and 10 (37.0%) patients presented a FOIS ≥6 (Table 2).

For the second aim of this study, univariable regression analysis revealed a significantly increased risk for moderate/severe OD in patients with FOIS 1–5 (OR 17.0, 95%CI 1.885 - 153.273). This increased risk remained significant after correction for age (OR 22.3, 95%CI 2.138–233.346) or BMI (OR 14.126, 95%CI 1.525–130.831) in multivariable regression analysis (Table 3).

**Table 3:** Univariable and multivariable regression analyses to assess the relationship between OD severity (DSS) and respectively functional oral intake (FOIS) and patients' dysphagia-specific QoL (MDADI).

**Univariable analyses**									
	***n***	**OR**	**95% CI**	***P*-value**	**Nagelkerke R^2^**				
No relevant OD^a^/mild OD	*Reference*								
Moderate/severe OD									
*FOIS^*b*^ 1*–*5*	*38*	17.0	(1.885–153.276)	0.012*	0.339				
*MDADI^*c*^-total*	*36*	0.956	(0.907–1.008)	0.098	0.119				
*MDADI-global*	*38*	0.683	(0.395–1.182)	0.173	0.071				
*MDADI-functional*	*38*	0.839	(0.697–1.01)	0.064	0.144				
*MDADI-physical*	*40*	0.978	(0.899–1.078)	0.653	0.007				
*MDADI-emotional*	*39*	0.863	(0.723–1.03)	0.103	0.107				
**Multivariable analyses**									
**Confounding factor**	**Age**					**BMI**			
	***n***	**OR**	**95% CI**	***P*** **-value**	**Nagelkerke R** ^2^	**OR**	**95% CI**	***P*** **-value**	**Nagelkerke R** ^2^
No relevant OD/mild OD	*Reference*					*Reference*			
Moderate/severe OD									
*FOIS 1-5*	*38*	22.334	(2138–233.346)	0.009*	0.357	14.126	(1.525–130.831)	0.020*	0.355
*MDADI-total*	*36*	0.951	(0.900–1.006)	0.079	0.137	0.959	(0.908–1.014)	0.143	0.123
*MDADI-global*	*38*	0.679	(0.392–1.177)	0.168	0.075	0.732	(0.421–1.275)	0.271	0.079
*MDADI-functional*	*38*	0.834	(0.689–1.010)	0.063	0.146	0.852	(0.703–1.032)	0.102	0.139
*MDADI-physical*	*40*	0.979	(0.887–1.081)	0.680	0.008	0.990	(0.896–1.095)	0.851	0.029
*MDADI-emotional*	*39*	0.858	(0.717–1.028)	0.097	0.111	0.860	(0.716–1.032)	0.105	0.144

^*a*^Oropharyngeal Dysphagia (OD).

^*b*^Functional Oral Intake Scale (FOIS).

^*c*^MD Anderson Dysphagia Inventory (MDADI).

*p < 0.05.

Median MDADI subdomain scores did not significantly differ between the DSS groups (Table 2). Univariable and multivariable regression analyses did not show a significant relationship between DSS scores and MDADI subdomain scores (Table 3).

## Discussion

This exploratory cross-sectional study reported specifically on patients with chronic post-stroke OD who were referred to the interdisciplinary outpatient clinic for OD. The patients' risk of malnutrition, the characterization and severity of OD, and dysphagia-specific QoL were the main points of attention. The study revealed that approximately two out of five patients with chronic post-stroke OD who visited the outpatient clinic, had a moderate to high risk of malnutrition. More than half of the patients in the total population were moderately to severely dysphagic. In the high-risk malnutrition group, almost four out of five patients had a moderate to severe degree of OD. These results reinforce the need of screening for the risk of malnutrition in patients with chronic post-stroke OD and vice versa.

Screening and diagnostics of malnutrition and OD and referral to expert healthcare professionals is important in stroke patients. Subsequently, a tailored treatment plan for the identified condition(s) can be developed by an expert healthcare professional. This may prevent poor clinical outcomes related to malnutrition or OD such as skeletal muscle mass loss, nutrient deficiencies, immune deterioration, and aspiration pneumonia. The majority of the patients in the present study were referred by a speech-and-language therapist from the primary healthcare network to the interdisciplinary outpatient clinic for OD. The speech-and-language therapist of the primary healthcare network indicated the referral based on a clinical swallow assessment without having access to imaging techniques such as among others, FEES or videofluoroscopic swallow study. Based on the SNAQ, the standardized screening for malnutrition risk used in the outpatient clinic, the patient could immediately be referred to the dietitian who provided additional information relevant to tailor the treatment for OD.

Interestingly, almost half of the population had a moderate or high risk of malnutrition according to the SNAQ despite the relatively high average BMI values in this population. According to the commonly used cut-off values for BMI, thus in disregard of deviating cut-off values in specific patient populations and in older patients, the present study population could be classified as overweight (mean BMI 26.8 kg/m^2^). Malnutrition in overweight patients can easily be overlooked based solely on appearance or body posture. In the present study, the BMI values did not differ between the patient group with low malnutrition risk vs. the patient group with moderate/high malnutrition risk. Similar BMI values were found in the patient group that used ONS and/or tube feeding and in the patient group that did not use ONS and/or tube feeding. European wide, similar BMI values were seen in stroke patients (77). The mean (±SD) BMI of 198 stroke patients from seven European countries was 26.9 (±4.9) kg/m^2^. These patients were assessed between three and 12 months post-stroke and 19.7% of these patients had a BMI ≥30 kg/m^2^. Stroke may be associated with a pre-stroke sedentary lifestyle (28, 78) affecting the patients' weight prior to the stroke event. BMI values from the present study may suggest that patients from this specific sample of referred stroke patients were not energy deprived, but instead, the SNAQ score showed the opposite. Thus, these patients may also be nutrient deficient and compliance of intake requires attention to minimize the risk of qualitative undernutrition with a normal BMI. Therefore, repeated screening for malnutrition and monitoring the severity and management of OD remain important.

BMI is sensitive to weight changes and age-related physiological changes and does not provide any information on potential nutritional deficiencies or body composition (i.e., sarcopenia). Nutritional screening based on a valid comprehensive screening tool such as the SNAQ is recommended as it encompasses multiple indicators for malnutrition besides BMI (79).

A consensus on criteria to screen and assess the patients' nutritional status was lacking until recently. Existing examination tools were not always validated and/or were mistakenly used interchangeable (80). In 2019, consensus criteria for the diagnosis of malnutrition, the Criteria for the Diagnosis of Malnutrition (GLIM), were published (81, 82). At that point and during the current study period the outpatient clinic used the SNAQ as part of the standard swallow protocol to screen for malnutrition risk. The SNAQ is a validated, time-efficient, and non-invasive screening tool in the outpatient clinic (63). The SNAQ score provides an indication of the risk of malnutrition and is useful in the referral of patients to a dietician. Patients in the present study who scored high on the SNAQ were indeed referred to a dietician, and it was shown that more than one third of the population received ONS or tube feeding, and more than half of the population was treated for post-stroke OD by the speech-and-language therapist. These results suggested that post-stroke healthcare in the Netherlands is already pro-actively targeting OD rehabilitation and nutritional interventions in these patients, including initiation of ONS or tube feeding. This is in accordance with the ESPEN guidelines for clinical nutrition in neurology (83). The Netherlands has been top ranked on the Euro Health Consumer Index (EHCI) continuously (84). Moreover, ONS or tube feeding and/or speech-and-language therapy are reimbursed by the Dutch health insurance system and an extensive primary healthcare network of (allied) health professionals providing nutritional- and OD care, exists (85).

Despite these positive care-related aspects, there was still a high prevalence of patients visiting the outpatient clinic who had a moderate/high risk of malnutrition. The present study showed that the prevalence of patients with unintentional weight loss was higher among patients in the high-risk group as compared to patients in the low-risk of malnutrition group. Almost three out of four patients in the high-risk group experienced unintentionally weight loss >6 kg in 6 months.

This raised the question whether these patients were undertreated from a nutritional perspective. Based on the results from the present study this question remained unanswered as specific details on nutritional interventions and OD treatment, e.g., type of intervention, standardization and duration of treatment, were limited to the use of ONS or tube feeding and the presence or absence of speech-and-language therapy.

As a previous literature review showed some evidence of a relationship between the presence of OD and the risk of malnutrition in stroke patients (86), the present exploratory study aimed to increase the body of evidence on this relationship by investigating a well-defined high-risk patient subgroup with chronic post-stroke OD. The severity of OD did not seem to play a role in the risk of malnutrition in the present study population, as no significant difference in DSS score was found between the low vs. high-risk of malnutrition groups. However, the sample size may have been too small to find such a relation, since interestingly more patients with severe OD were present in the high-risk malnutrition group (47.1%) vs. the low-risk group (22.7%).

Additional analyses revealed that the severity of OD was significantly related to the level of functional oral intake in these patients, but the wide confidence intervals indicated that the interpretation of this relationship requires caution. A relationship between OD severity and the patients' dysphagia-specific QoL could not be confirmed in the present small study sample. The chronic state of OD of these patients may have led to an acceptance of function loss or patients may have relatively more severe complaints that affect health-related QoL, such as loss of independence, or absent sexual relationships.

Regression analysis did not show a significant relationship between OD severity and the patients' dysphagia-specific QoL. However, a significant relationship between the severity of OD and impaired functional oral intake was found.

Considering the available literature on nutritional status in chronic stroke patients and the relationship between nutritional risk and the severity of OD in the present study, it is probably insufficiently known that many chronic stroke patients simultaneously suffer from chronic OD and are at risk of malnutrition. Current (inter)national clinical guidelines for stroke care recommend a screening for OD and malnutrition in the acute phase and proper management of these conditions (83, 87). There is however a lack of attention for these conditions on the longer term. Future research in this field is recommended as stroke survivors will age and their functionality may deteriorate which makes the implementation of repeated screening for OD and nutritional risk in the chronic phase after stroke an important point of attention. Repeated screening for both health problems, OD and nutritional risk is recommended as correlations between OD and nutritional risk have not been found in the present study despite the fact that both health problems are highly prevalent. One could think of a long-term surveillance plan which includes repeated screening for OD and nutritional risk at preset time points over the course of 5 years after stroke, as recommended in head and neck cancer patients (88). In the Netherlands, this could be integrated in the repeated follow-up for cardiovascular risk management in persons 60+ years of age at the general practitioner (89). Repeated follow-up visits may also provide insights in the impact of stroke on functionality at older age.

### Study limitations

The present study was an exploratory cross-sectional study in which valid assessment methods were used to describe nutritional risk and characteristics, and severity of OD in a group of patients with chronic post-stroke OD. Nonetheless, this exploratory study design brought along some limitations.

Some statistically significant results were found in the present study, although the sample size was too small to uncover all relevant associations. Wide confidence intervals from regression analyses showed that the interpretation requires caution. However, the patient population of the present exploratory cross-sectional study was a realistic representation of patients with chronic post-stroke OD consulting the interdisciplinary outpatient clinic for OD in a tertiary university referral hospital in the Netherlands.

Furthermore, the interpretation of severity scores for OD such as the DSS requires caution. There is no consensus in the literature on what would be the optimal scale for the classification of the severity of OD. For the present study we applied the best viable option at this moment being the Dysphagia Severity Scale. The DSS was based on the presence or absence of premature spillage and/or pharyngeal residue, penetration or aspiration events of one or multiple bolus consistencies, but did not take ‘other signs' of OD such as pre-swallow loss of bolus into the pharynx, clearing swallows, etc. into account. The lowest DSS score did not indicate total absence of OD, as ‘other signs' of OD may still have been present. Despite these limitations, the DSS was used as OD severity scale for the present study based on the FEES-registry study showing clinical relevance of this scale and a positive correlation with the FOIS in a very large multicenter study including 2,401 patients with neurogenic OD of whom 1,465 had post-stroke OD (75). Finally, FEES is a reliable and feasible test method to evaluate the pharyngeal phase of swallowing in stroke patients. Disturbances in the pre-oral and oral phase of swallowing may affect nutritional intake, though these disturbances cannot be visualized using FEES.

## Conclusion

This exploratory cross-sectional study showed that more than half of the patients from the present specific sample of referred stroke patients had moderate to severe OD and approximately half of this population also had a moderate to high risk of malnutrition. Despite the use of clinical practice guidelines on stroke and a normal nutritional status at first sight, repeated screening for malnutrition and monitoring the severity and management of OD remain important elements in the care of patients with chronic post-stroke OD.

## Data availability statement

The data analyzed in this study is subject to the following licenses/restrictions: The dataset analyzed during the current study are not publicly available due to privacy restrictions. The data are available from the corresponding author on reasonable request under the condition that the data privacy of participants is not compromised. Requests to access these datasets should be directed to vivienne.huppertz@gmail.com.

## Ethics statement

The studies involving human participants were reviewed and approved by the Medical Ethics Committee of the University Hospital Maastricht and Maastricht University (METC azM/UM). Written informed consent for participation was not required for this study in accordance with the National Legislation and the Institutional requirements.

## Author contributions

Material preparations, data collection and analyses were performed by VH, WP, GP, and LB. The first draft of the manuscript was written by VH, WP, and LB. All authors commented on previous versions of the manuscript, contributed to the article, and approved the final manuscript.

## Funding

Support for this work has been received from Maastricht University, Maastricht, Netherlands and from Danone Nutricia Research, Utrecht, Netherlands. The funders were not involved in the study design, collection, analysis, interpretation of data, or the decision to submit it for publication.

## Conflict of interest

AH discloses to be an employee of Danone Nutricia Research. VH and JS receive financial support for their research from Danone Nutricia Research. LB is a consultant for Phagenesis Limited. The remaining authors declare that the research was conducted in the absence of any commercial or financial relationships that could be construed as a potential conflict of interest.

## Publisher's note

All claims expressed in this article are solely those of the authors and do not necessarily represent those of their affiliated organizations, or those of the publisher, the editors and the reviewers. Any product that may be evaluated in this article, or claim that may be made by its manufacturer, is not guaranteed or endorsed by the publisher.
