# Supplementary material for: Malnutrition risk and oropharyngeal dysphagia in the chronic post-stroke phase
Source: Front Neurol. 2022 Sep 28;13:939735. doi: 10.3389/fneur.2022.939735 (PMC9554502; doi:10.3389/fneur.2022.939735)
Supplement: Supplementary file 1 [file Table_1.pdf]

Table S1 Description of the fiberoptic endoscopic evaluation of swallowing variables

**FEES[a] variable**	**Description**	**Scale**
Penetration-aspiration	Entry of the bolus into the larynx, remaining on or above the vocal folds (penetration) or below the level of the vocal folds (aspiration). Bolus on the true vocal folds or in the anterior commissure secondarily leaking in the trachea was also classified as aspiration.	Trichotomous scale (range 0–2)<br>0 = no penetration or aspiration<br>1 = penetration<br>2= aspiration
Pharyngeal residue	Bolus remaining in the pharynx after spontaneous clearing swallows. No distinction was made between right- or left-sided residue.	Dichotomous scale (range 0–1)<br>0 = no residue<br>1 = residue
Other signs of OD[b]	Pre-swallow loss of bolus into the pharynx (pre-swallow posterior spill), delayed initiation of the pharyngeal reflex, and/or clearing or repeated swallows on the same bolus (piecemeal deglutition).	Dichotomous scale (range 0–1)<br>0 = no other signs of OD<br>1 = other signs of OD
Dysphagia Severity Scale (DSS)	Severity of OD based on the absence or presence of premature spillage and/or residue, penetration or aspiration events and on the number of consistencies at which the penetration or aspiration events occurred.	4-point scale (range 0-3)<br>0 = no relevant dysphagia<br>1 = mild dysphagia (premature spillage and/or residue, but no penetration-aspiration events)<br>2 = moderate dysphagia (penetration-aspiration events with one consistency)<br>3 = severe dysphagia (penetration-aspiration events with two or more consistencies)

[a] Fiberoptic endoscopic evaluation of swallowing (FEES)
[b] Oropharyngeal dysphagia (OD)
